# Where ecologically ‘tis better to go brown than green: enhanced seagrass macrobenthic biodiversity within the canals of a brownfield coastal marina

**DOI:** 10.1007/s10531-022-02468-9

**Published:** 2022-08-11

**Authors:** Richard S.K Barnes, Louw Claassens, Jessica Seath

**Affiliations:** 1 Department of Zoology and Entomology, Rhodes University, 6140 Makhanda, Eastern Cape Republic of South Africa; 2 Department of Zoology and Conservation Research Institute, University of Cambridge, Cambridge, UK; 3 Present Address: Palau International Coral Reef Center, 96940 Koror, Republic of Palau

**Keywords:** Brownfield, Knysna, Marina, Seagrass, Subtidal macrobenthos

## Abstract

At the start of the 21st century, a coastal residential-estate marina was developed on a previously degraded and polluted brownfield island site within Knysna estuarine bay, Garden Route National Park, South Africa, including the creation of 25 ha of new flow-through tidal canals. Canals near the larger entrance to this system now support permanently submerged beds of seagrass, which in turn support abundant macrobenthic invertebrates. In comparison with equivalent seagrass-associated assemblages present in natural channels around the island, those in the artificial marina canals were similarly structured and dominated by the same species, but the marina assemblages were significantly more species-rich (1.4 x on average) and were more abundant. Indeed, this area of marina supports the richest seagrass-associated macrofaunal biodiversity yet recorded from South Africa. The canals created de novo therefore now form a valuable addition to the bay’s marine habitat, in marked contrast to the generality that marinas developed on greenfield sites represent a net reduction in intertidal and shallow marine area and associated seagrass-associated benthos. If located and constructed appropriately, brownfield marina development and conservation of coastal marine biodiversity clearly need not be antithetical, and brownfield sites may provide opportunity for the location and management of ‘artificial marine micro-reserves’ or for the action of ‘other effective area-based conservation measures’ for soft-sediment faunas.

## Introduction

Marine coastal systems are becoming increasingly urbanised (Airoldi et al. 2008; Bishop et al. 2017), and this development includes the many residential marina estates that have appeared along sheltered stretches of the world’s coastline over the last few decades (Waltham and Connolly 2011). Indeed “there is barely a waterfront city or community in the developed world that doesn’t have a marina … [and] entire self-contained cities … [have] been developed around a new harbour and marina complex” (Natchez 2018). Many more are proposed, several in areas of shore and shallow sea notionally protected by Ramsar, Marine Park or equivalent conservation declarations (Sheppard 2019). It is estimated that between 1984 and 2016 16% of the world’s intertidal-flat habitat was lost to a range of causes, predominant among which was coastal development (Murray et al. 2019, 2022).

Although the negative effects of marina construction, especially on shorebirds (e.g. Duggan et al. 2011; Bishop et al. 2017), the spread of non-native species (Bulleri and Chapman 2010; Foster et al. 2016), and water quality (Rivero et al. 2013; Valdor et al. 2019), have caused widespread conservation concern, research on the endangered estuarine seahorse, *Hippocampus capensis*, has shown that this species has benefitted from the suitable new subtidal habitat provided by marina canals (Claassens 2016; Claassens and Hodgson 2018). A positive effect of marinas on the survival of juvenile native seabreams, *Diplodus* spp, along the French Mediterranean coast has also been suggested (Bouchoucha et al. 2016), although this may be an artifact of acting as attractors of juvenile fish (Bosch et al. 2017). The impact of coastal marinas has generated a large volume of research but relatively little of this has concerned their effect on soft-sediment benthic invertebrates and effectively none on seagrass macrobenthos or on the new aquatic habitats constructed on brownfield sites (i.e. on previously developed land no longer in use); although unsurprisingly it is known that hard surfaces are a ‘poor substitute’ for natural soft-sediment habitats including seagrass beds (Momota and Hosokawa 2021).

Thesen Islands Marina is one such 96 ha artificial residential-estate complex located within the marine embayment section of the warm-temperate Knysna estuarine bay, part of South Africa’s Garden Route National Park. It was constructed on a brownfield terrestrial site in the early 21st century (2000–2007) and includes some 25 ha of flow-through tidal canals, dug *de novo* across a former bay island and lined by gabions and floored by Reno mattress® (Claassens 2016). As Waltham and Connolly (2011) emphasised, research on the value of marina-associated waterways as aquatic habitats can and should underpin planning and management of these systems. Therefore to address this lack, the present study extended earlier work carried out on the macrobenthic invertebrate biodiversity associated with subtidal seagrass along the axial channel at Knysna (Barnes and Claassens 2020) by comparing this natural *Zostera capensis* system to that associated with the same species in the adjacent, recently-developed area of the artificial Thesen marina-canal system. The study examined the hypothesis that the benthic invertebrate fauna supported by the *Zostera* in the artificial marina-canals is an impoverished version of that present in natural beds of the same seagrass in the channels surrounding the marina, both in terms of abundance and biodiversity.

## Methods

**Fig. 1:**
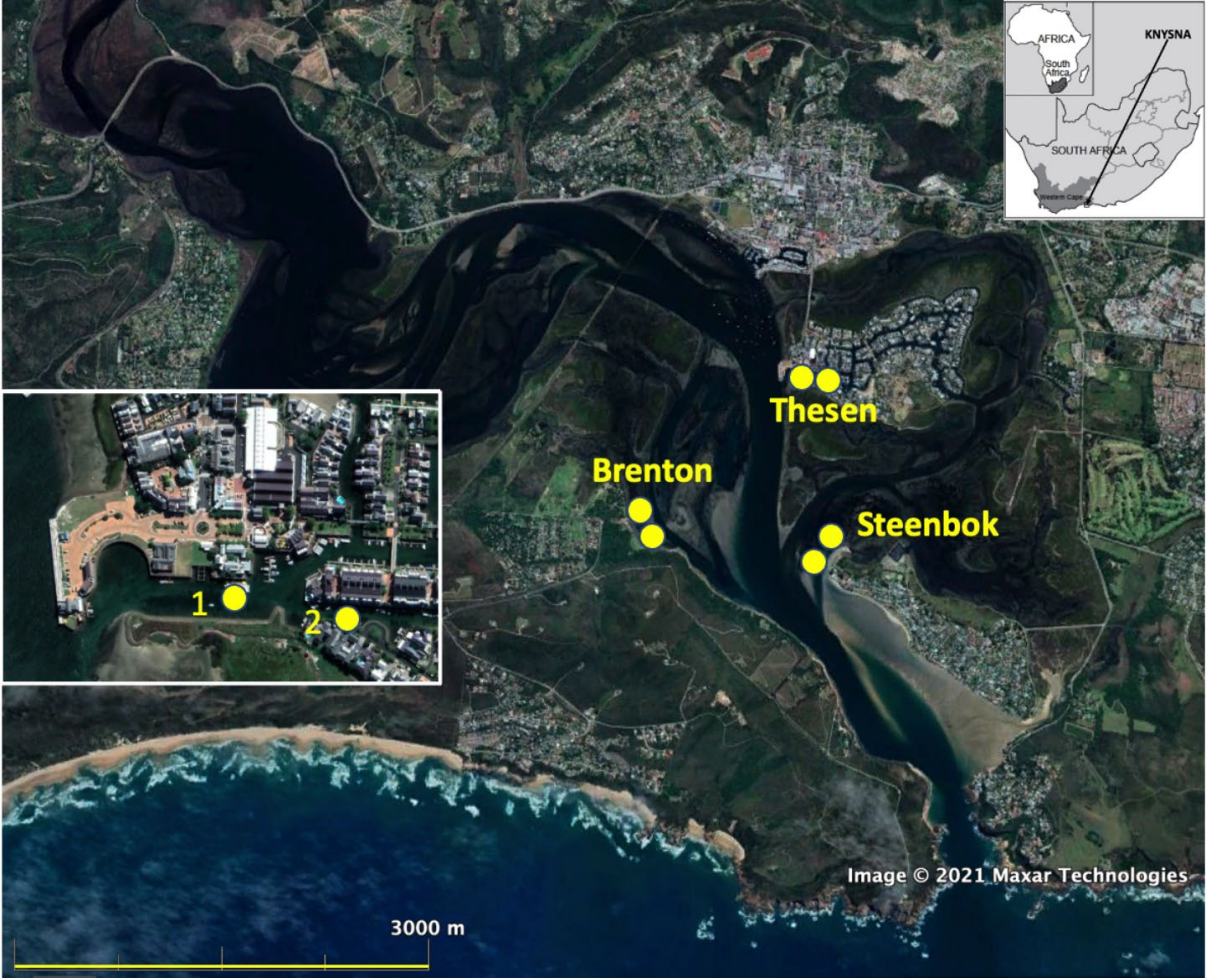
Knysna estuarine bay (Google Earth image), showing the location of sampling sites in the natural *Zostera*-floored channels near Thesen Island (‘Brenton’ and ‘Steenbok’) and those in the artificial canals (‘Thesen’) of the Thesen Islands residential marina estate (location of the canal sites shown in greater detail in box)

Macrofaunal sampling in the Thesen canals was conducted by snorkelling mainly during the 2020 austral summer, the research being approved by SANParks and conducted in accordance with their scientific research regulations and requirements. Two sites, *c.* 100 m apart, near the western entrance known to support *Zostera* (Claassens 2016) (at 34º02’58.1"S.23º02’51.7"E & 34º02’59.3"S.23º02’57.7"E, Fig. 1) were sampled at a depth of some 2 m below LWS using identical procedures to those described earlier for the natural subtidal sites along the Knysna axial channel (Barnes and Claassens 2020), i.e. each site by 16 cores each of 0.0054 m^2^ area and 100 mm depth. For one of the two sites, the onset of covid-19 related restrictions on travel and fieldwork prevented completion of sample collection in 2020, and the remaining cores were taken in summer 2022: species density values were lower in 2022 than in 2020 although the difference was not significant (ANOVA *F*_1,14_ = 1.9; *P* = 0.08).

Cores were gently sieved (‘puddled’) through 710 µm mesh on site. This sampling procedure collects the smaller and more numerous members of the macrofauna that constitute the large majority of invertebrate biodiversity (Bouchet et al. 2002; Albano et al. 2011), though not the meiofauna nor much scarcer megafauna nor sessile animals attached to the seagrass leaves. Warwick et al. (2006) have shown that different patterning rules may apply to meiofauna and macrofauna, and likewise Davidson et al. (2004) and Leopardas et al. (2014) to sessile species. Sessile or semi-sessile species such as *Siphonaria compressa* and *Anomia achaeus* that had accidentally become detached from the seagrass leaves during sampling were therefore ignored. Animals were identified to species level wherever possible, with all organismal nomenclature being as listed in the World Register of Marine Species (www.marinespecies.org) accessed December 2021, except in respect of the unlisted ‘*Assiminea*’ *capensis* (see Barnes 2018). It should be noted, however, that the specific identity of several animals, especially amongst the Polychaeta, is questionable because of lack of recent revision; those of South African taxa of Polycladida, Oligochaeta and Nemertini, and many members of other groups less than 3–4 mm in largest dimension, are virtually unknown. Such animals were treated as morphospecies, an operationally appropriate procedure to detect spatial patterns of numbers of species and their differential abundance (Dethier and Schoch 2006; Gerwing et al. 2020).

## Analyses

Macrobenthic assemblage data from the marina-canal *Zostera* were compared with the equivalent relevant data for the macrobenthic assemblages in natural subtidal channels obtained by Barnes and Claassens (2020) in the same summer of 2020, i.e. those located at the nearby Brenton and Steenbok localities at similar depth below LWS (Fig. 1). The Steenbok locality was in the southern mouth of the Ashmead Channel that separates Thesen Island from both the mainland and from the nearby Leisure Isle; it was floored by clean tidal-delta sand and subject to strong water flow. That at Brenton was more sheltered and was floored by muddy sand. All three localities are fully marine and bathed by semidiurnal fluxes of water from the Indian Ocean. Numbers of each component zoobenthic taxon at the natural and artificial seagrass sites were subjected to similarity analysis, and assemblage metrics per unit area were derived and compared via *PAST* 4.0.9 software (Hammer et al. 2019), all based on abundance data (Beck et al. 2013).

Univariate metrics assessed were those known to have a major influence on local-scale biodiversity patterns (Blowes et al. 2022); i.e. (i) overall faunal numbers per unit area, (ii) observed numbers of taxa per unit area, *N*_0_ [i.e. ‘species density’ *sensu* (Gotelli and Colwell 2001)], and (iii) relative evenness (= equitability) assessed as Pielou’s *J*. Estimates of likely total number of taxa per unit area were obtained via Chao1 and ACE (abundance-based coverage estimation) values. In addition, and as previously at Knysna (Barnes 2019), patchiness in spatial abundance of the macrofaunal associations at each site and station was also quantified by spatial point pattern analysis using Lloyd’s (1967) index of patchiness, *I*_p_. Multivariate comparison of macrofaunal assemblage composition used hierarchical clustering analysis of S_17_ Bray-Curtis similarity (Legendre and Legendre 1998), ANOSIM, PERMANOVA and SIMPER, carried out with 9999 permutations. Differentials between the structure of artificial marina-canal and natural-channel assemblages were also assessed by comparison of ranked taxon index of numerical importance (INI) distributions, analogous to species abundance distributions (Antão et al. 2021) but derived from both abundance and occupancy data (Barnes 2014). For this, all data sets were standardised for overall taxon density (by dividing all ranks by the total number of taxa in the set) and for sample size (by dividing the abundance of each taxon by the overall number of individuals in the set) (Passy 2016). Curves were compared using one-way ANCOVA, and differences in number of taxa and of individuals per core between the three localities were tested by one-way ANOVA and post hoc Tukey HSD tests.

## Results

In total, the 96 subtidal samples yielded 16,407 animals of 116 taxa at an overall density of 31,500 m^− 2^, of which gastropod molluscs comprised 88% and the microphytobenthically-feeding cerithioid microgastropod *Alaba pinnae* 82%. The 32 samples from the marina canals totalled 6,870 individuals of 83 taxa from a total area of 0.17 m^2^; a local density of 39,757 m^2^ — these values constitute the most biodiverse fauna of any South African *Zostera* bed, and estimates of the true number of taxa in the sampled area at Thesen are considerably larger still at Chao1 = 129 and ACE = 121, one third of the taxa being singletons. The 64 natural-channel samples yielded a total of 86 taxa and a smaller estimates of likely total numbers, i.e. Chao1 = 109 and ACE = 108, with similar proportions of singletons (30–34%). Even though only formed less than 20 years ago, the marina *Zostera* beds therefore supported more macrobenthic species per unit area than either of the two natural channels investigated (1.38 x as many per core sample on average) (ANOVA *F*_2,93_ = 29; Tukey HSD *P* < < 0.0001) (Table 1). Core samples from the marina canals also yielded 1.44 x as many individual animals as the average for the natural channel sites, although this difference was (marginally) not significant (ANOVA *F*_2,93_ = 2.87; *P* = 0.06). The difference in numbers of non-*Alaba* individuals, however, was highly significant (ANOVA *F*_2,93_ = 22; *P* < < 0.0001; all Tukey HSD *P* < 0.02); whereas those in *Alaba* numbers was not (ANOVA *F*_2,93_ = 1.2; *P* = 0.3).

**Table 1:** Metrics of the macrobenthic invertebrate assemblages of *Zostera capensis* beds in the six sites investigated within the marine embayment of the Knysna estuarine bay (means ± S.E.). Numbers of taxa per unit area at Thesen differ markedly from those at both Brenton and Steenbok (Tukey *P* < 0.00001), and those in the two natural channels also differ although more marginally (*P* = 0.047); numbers of individuals differ between Thesen and Steenbok (*P* < 0.05) but not between other localities (*P* > 0.4)

	Brenton		Steenbok		Thesen	
	1	2	1	2	1	2
Nos total ind. core^− 1^	188 ± 41	158 ± 20	29 ± 4	221 ± 27	217 ± 35	212 ± 58
Nos *Alaba* core^− 1^	149 ± 42	132 ± 20	11 ± 2	209 ± 28	165 ± 35	173 ± 58
Nos taxa core^− 1^	12 ± 1	11 ± 1	10 ± 1	9 ± 1	17 ± 1	15 ± 1
Nos taxa 0.09 m^− 2^	40	41	47	40	58	61
Evenness (*J*)	0.27	0.24	0.67	0.10	0.30	0.26
Patchiness (*I*_p_)	1.71	1.23	1.21	1.23	1.38	2.13

The seagrass-associated species concerned were mostly classic members of the southern African estuarine fauna as identified by Day (1981) and de Villiers et al. (1999), but the superabundance of the *Zostera*-leaf associated *Alaba pinnae* appears most unusual. *Alaba* is also present, though by no means dominant, in the nearby Swartvlei estuary to the west (Whitfield 1989) but has not been recorded by surveys of other South African estuaries (Barnes and Claassens 2020), except sporadically in the St Lucia / iSimangaliso system (Perissinotto et al. 2014), and apparently it does not occur in the Keurbooms/Bitou Estuary immediately to the east of Knysna (Duvenage and Morant 1984; Villiers et al. 2021). Leaving aside the overwhelmingly dominant *Alaba*, with the exception of a single core sample containing large numbers of small nereid worms, compared to the natural channels the Thesen canals supported proportionately fewer polychaete individuals (11% of the total vs. 20%) and those of other gastropod taxa (28% vs. 37%), but more crustaceans (48% vs. 37%), bivalves (5% vs. 2%) and echinoderms (3% vs. 1%) (see Supplementary data). These relative abundances of polychaetes, crustaceans and echinoderms are the same as those of the MLW → LWS → subtidal gradient at Brenton and Steenbok (from data of Barnes and Claassens 2020), although this may be purely coincidental.

Excluding rarities, no species common to the two natural channels was noticeably absent from the Thesen canals, nor was any species present there not also represented in the natural channels, except the small bivalve *Gafrarium pectinatum* which occurred in the canals at a density of 214 m^− 2^, although the amphipods *Ericthonius* and *Monocorophium*, and the gastropod *Gibbula* were at least six times more abundant in the *Zostera* of the canals than outside the marina. The two amphipods *Ericthonius* and *Monocorophium* are certainly aliens or cryptogenics (Griffiths et al. 2009), as are the amphipod *Jassa* and isopod *Paracerceis*: all four are now distributed right across the globe [Global Biodiversity Information Facility (www.gbif.org), accessed February 2022]. *Erichthonius* and *Jassa* were present in Knysna before 1950 (Day et al. 1951), whilst the other two are perhaps more recent arrivals, although they also predate the Thesen marina. Both are widespread along the South African coast having been distributed in association with oyster cultivation and via commercial shipping (Griffiths et al. 2011), two activities that have influenced the Knysna marina fauna for many decades.

Other notable occurrences in the canals included the rare and critically endangered, seagrass false-limpet *Siphonaria compressa* and the uncommon seagrass-associated form of the starfish *Parvulastra exigua* — both known only from two localities, both in the Western Cape (Knysna and the Langebaan estuarine lagoon). An *Elysia* amongst the collected material is almost certainly a new species (T. Gosliner pers com). The suites of numerically dominant species at the three localities are shown in Table 2.

**Fig. 2:**
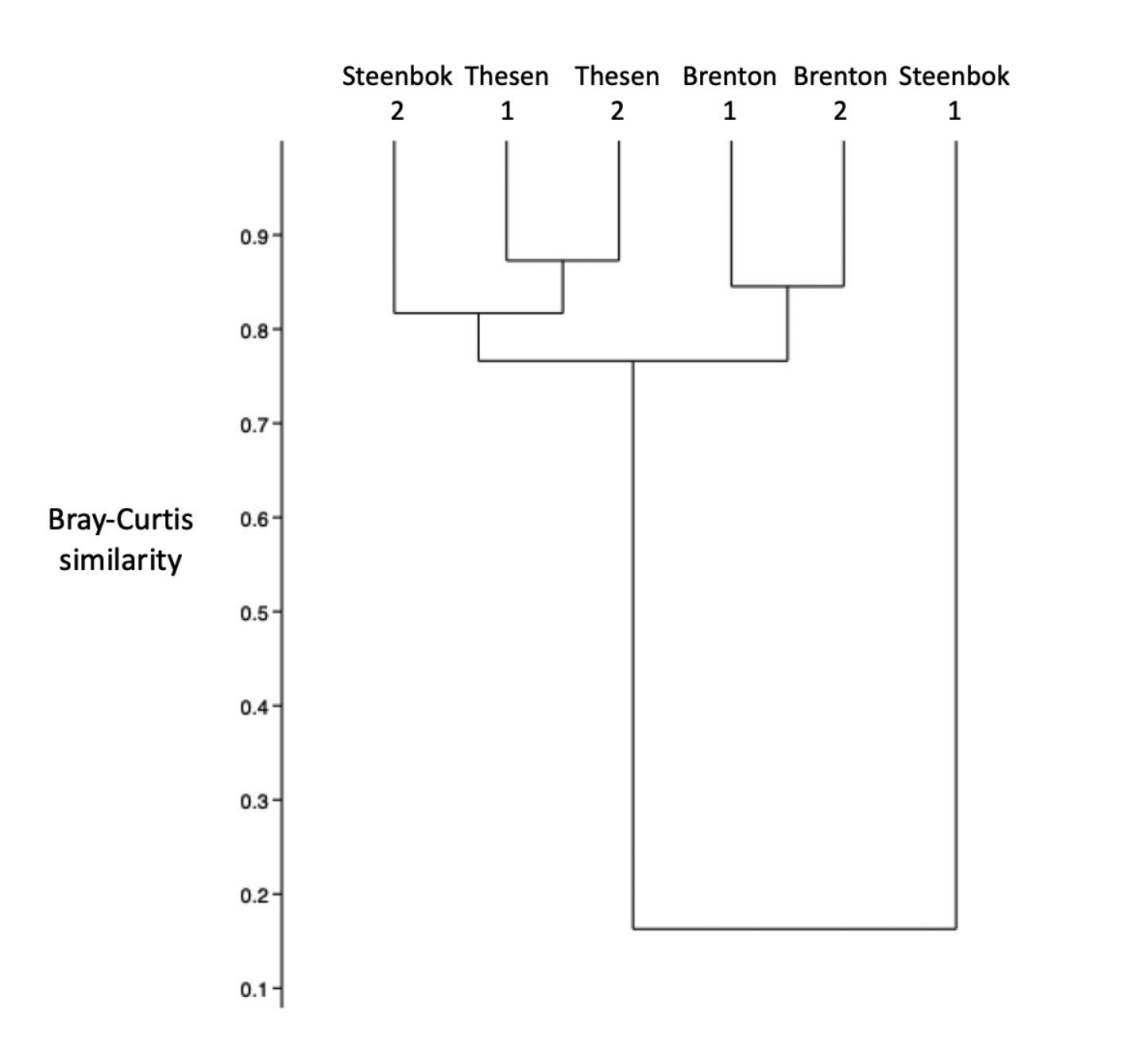
Bray-Curtis compositional similarity of the macrofaunal benthic assemblages associated with the seagrass *Zostera capensis* at the various artificial and natural sampling sites

Variation in the abundance of *Alaba* accounted for > 65% of the assemblage compositional differences between the three localities (SIMPER); all comparisons between individual sites yielding significant differences (ANOSIM *R* ≥0.12; *P* < 0.02; PERMANOVA *F* > 4; *P* < 0.008) except that between the two Brenton sites. At the level of the three localities, all three were significantly different (PERMANOVA *F* > 4.8; *P* < 0.003). Bray-Curtis similarities between the macrobenthic assemblages (Fig. 2) indicated that the artificial Thesen sites were nested within those of the two local natural channels; that between the natural- and artificial-channel assemblages being 0.73. The greater abundance of *Alaba*, *Gibbula, Ericthonius* and ?*Cylindroleberis*, and the smaller densities of ‘*Assiminea*’, *Turritella* and *Grandidierella*, in the Thesen canals accounted for > 80% of such differences between the artificial canals and natural channels as there were (SIMPER). To a large extent *Grandidierella* was replaced in the canals by another aorid ?*Bemlos* sp. that was more than twice as abundant there as in the natural channels.

**Table 2:** Rank order of the eight most numerous species (each comprising ≥1% of total individuals) associated with *Zostera capensis* at the three subtidal localities. Variation in local density of *Alaba* is the factor most responsible for differences in assemblage composition between the three localities, and, excluding that component, variation in abundance of the other top six species in the Table is the factor next most responsible. Species coding: ^1^epibenthic, ^2^ surface, ^3^ subsurface in the classification of Macdonald et al. (2010). Only the *Ericthonius* is a non-native species. See Supplementary Data for further information

	Brenton	Steenbok	Thesen
1.	*Alaba pinnae*^1^ (81%)	*Alaba pinnae*^1^ (88%)	*Alaba pinnae*^1^ (79%)
2.	'*Assiminea*' *capensis*^1,2^ (5%)	*Grandidierella lignorum*^2^ (1%)	*Gibbula cicer*^1^ (4%)
3.	*Turritella capensis*^3^ (3%)	*Paridotea ungulata*^1^ (1%)	*Ericthonius brasiliensis*^1^ (3%)
4.	? *Cylindroleberis* sp.^1^ (2%)	? *Cylindroleberis* sp.^1^ (2%)	? *Cylindroleberis* sp.^1^ (2%)
5.	*Grandidierella lignorum*^2^ (1%)	*Ericthonius brasiliensis*^1^ (1%)	*Nassarius kraussianus*^1,2^ (1%)
6.	*Pseudofabriciola* sp.^3^ (1%)	*Gibbula cicer*^1^ (1%)	aorid sp.^2^ (c.f. *Bemlos*) (1%)
7.	*Nassarius kraussianus*^1,2^ (1%)	*Hymenosoma orbiculare*^1,2^ (1%)	*Scoletoma tetraura*^3^ (1%)
8.	*Paradoneis lyra*^3^ (1%)	tubificid sp.^3^ (1%)	*Paratylodiplax algoense*^2^ (1%)

**Fig. 3:**
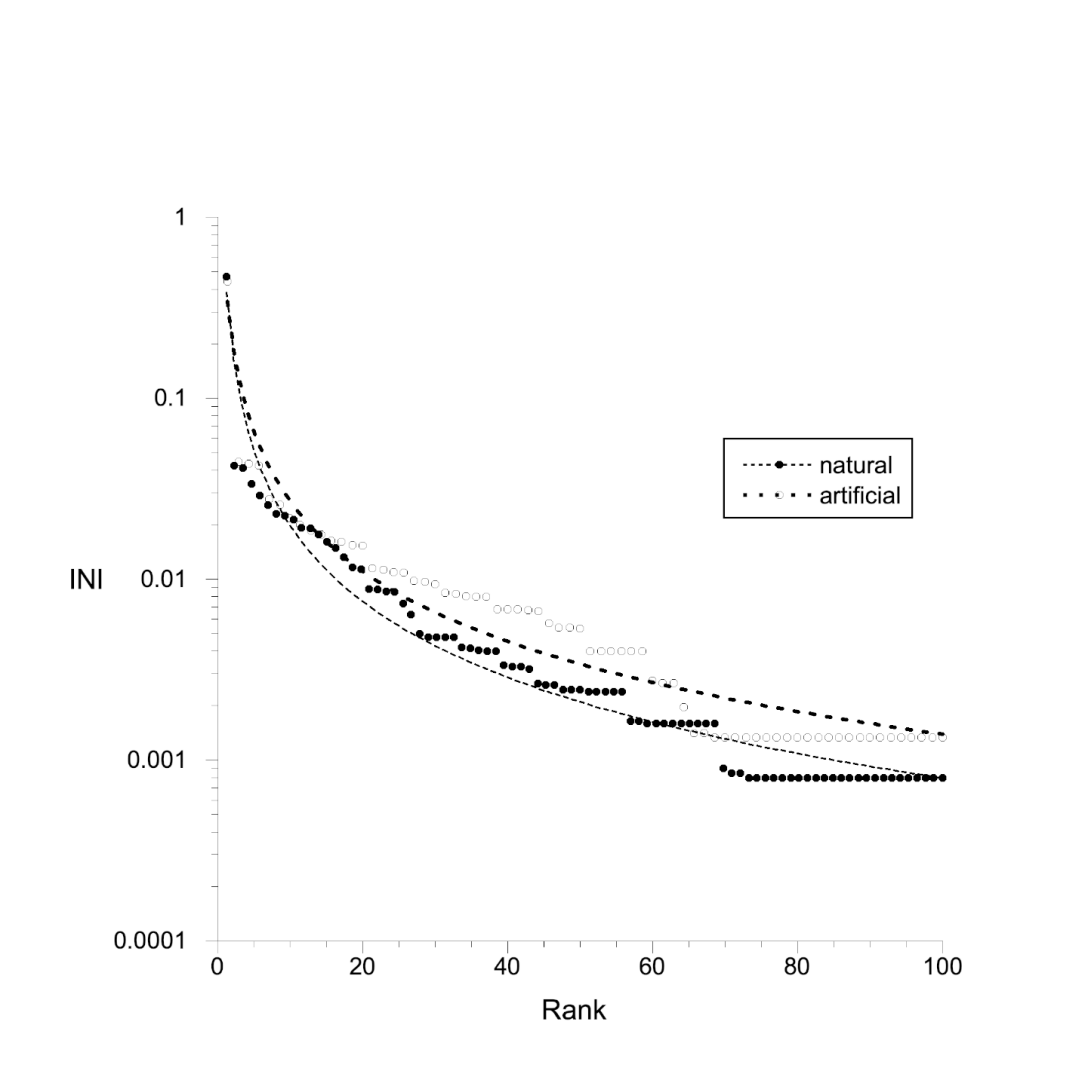
Curves of standardised ranked index of numerical importance for the subtidal macrobenthic assemblages in the artificial canals of the Thesen marina and in the natural channels adjacent to it

Assemblages values of evenness in the artificial canals were within the natural-channel range, and there was no significant difference between the ranked index of numerical importance curves for the assemblages in the natural channels and the artificial canals (ANCOVA *F* = 0.02; *P*_same_ = 0.89 for both mean values and slopes; Fig. 3). Values of patchiness were all considerably greater and more variable than those noted earlier for Knysna intertidal seagrass macrobenthic assemblages (Barnes 2019), mainly consequent on the large range in density of *Alaba* (2-868 per core; mean 140 ± 15 S.E.; *I*_p_ = 1.8–2.3; *P* < 0.0001).

In summary, the area within the larger entrance to the artificial marina-canal system of the Thesen Islands township, an area set aside and approved for development, therefore supported a greater abundance and greater biodiversity of seagrass-associated macrobenthos than did the *Zostera* in the natural channels of the Knysna marine embayment surrounding it, i.e. in those regions conserved as a Protected Environment under the South African National Environmental Management Protected Areas Act (No. 57 of 2003). Thus the tested hypothesis was disproved.

## Discussion

One of the known major influences of marinas, as of coastal urbanisation in general, is the addition of abundant hard surfaces to which sessile marine species can attach (Mineur et al. 2012; Rivero et al. 2013; Momota and Hosokawa 2021), including unwanted aliens. They also might be expected to impinge, however, on the ecology of areas of soft sediment, although from what little information appears to be available, it would seem that the benthic macrofauna of subtidal bare sediment is least affected by their presence. Thus Baird et al. (1981), in respect of an estuarine marina in the Eastern Cape, South Africa, and Chou et al. (2015), for three marinas in different stretches of Singapore’s coast, both found minimal consistent difference in faunal abundance and biodiversity inside and outside the marinas concerned, although the Singapore marina subject to least water flow experienced marked siltation and supported a reduced fauna dominated by a few opportunistic cirratulid and spionid polychaetes (see Maxted et al. 1997). Nevertheless, high levels of domestic and other pollutants can accumulate within the sediments of marina complexes (Guerra-García et al. 2021), as in other harbours (Guerra-García and García-Gómez 2005), and these may have consequences on faunal abundance and composition. Macrofaunas of areas covered by seagrass, however, are greatly affected; coastal development, including marina estates, being regarded as a major destroyer of seagrass beds and their inhabitants (Duarte et al. 2004).

Although many beds have been lost to concrete, there is evidence that seagrass systems can survive in close proximity to coastal development (Blake et al. 2014). At Knysna, this clearly includes *very* close to developed areas. The rich, *Alaba*-dominated, subtidal benthic macrofauna associated with *Zostera capensis* in the marine embayment section of the Knysna estuarine bay continues into the canal system of the brownfield Thesen marina with some increase in abundance of *Alaba* but especially with a significant increase in both abundance and species-richness of the associated fauna. Not all the canal system appears suitable for seagrass, however; indeed *Zostera* is present over less than 2% of the total area of marina waterways (Claassens 2016), being largely restricted to the high water-velocity region adjacent to the western entrance to the system. Areas with less current velocity support *Codium tenue* or mixed algal and seagrass communities (*Asparagopsis*, *Caulerpa*, *Halodule*, etc.) instead (Claassens 2016). But where it is present it grows luxuriously (leaf length > 1 m) and clearly supports a flourishing macrobenthos.

Knysna estuarine bay naturally supports the largest single area of the vulnerable species *Zostera capensis* in South Africa, which represents at least 25% and probably nearer 40% of the country’s total (Adams 2016). This seagrass covers nearly 40% of the whole Knysna system (Wasserman et al. 2020) and extends over a salinity gradient extending down to < 5 (Maree 2000). In this respect, distribution of *Z. capensis* at Knysna contrasts markedly with those of related *Zostera* spp (*marina* and *noltei*) which, at least in non-tidal lagoonal situations, have been described as being critically affected by salinity (positively) and nearness to sources of freshwater (negatively) (Boscutti et al. 2015). The height on the shore to which *Z. capensis* extends at Knysna is also relatively large: it routinely occurs right up to the interface with salt-marsh [0.2 m below HWN (Maree 2000)] and in some areas well into the fringing marsh vegetation (pers. obs.). Narrow, up-to-3 m-deep, marina canals flanked by tall buildings would not appear to be the ideal habitat for a permanently-submerged, light-loving seagrass (Lee et al. 2007), and together with its vertical and horizontal distribution this suggests that the Knysna population may be particularly tolerant of environmental extremes.

Knysna also supports the highest macrobenthic biodiversity of any known South African estuary (Turpie 2004; Turpie et al. 2004), containing some 40^+^% of that country’s estuarine total (Claassens et al. 2020); and within the Knysna system the most biodiverse region is its marine embayment wherein is located the Thesen marina (Barnes 2021). Clearly the construction of even marina estates on brownfield land within ‘nationally important’ areas of the ‘highest conservation priority’, as is the case with Knysna (Turpie et al. 2002; van Niekerk et al. 2019), is to say the least questionable (Barnes 1991; Airoldi and Beck 2007), not only because of the disturbance associated with the construction processes themselves (Iannuzzi et al. 1996; Prosser et al. 2018) and the danger of contamination (Leger et al. 2016), but also because of the effects thereafter of boat traffic (Sagerman et al. 2020; Carreño and Lloret 2021). In such a context, however, it is more than a little ironic that the region of highest *Zostera*-associated macrobenthic biodiversity within Knysna’s marine embayment, and hence in the whole of South Africa, is located in an artificial gabion-lined canal system, and that the canals also shelter two IUCN-listed endangered species. Although the seahorse *Hippocampus capensis* is often associated with *Zostera capensis*, the region of the Thesen canal system supporting the enhanced *Zostera*-associated invertebrate biodiversity is not the same one as those supporting enhanced densities of the endangered seahorse (Claassens 2016): the zone of increased invertebrate biodiversity near the mouth of the canal system is an additional conservation benefit to that of the seahorse population.

The Thesen marina system, however, is not likely to be typical of coastal marinas. Data are not readily available but personal experience suggests that almost all coastal marina estates have been constructed on greenfield marine sites (i.e. in previously natural habitat), the construction of which obliterates natural intertidal and subtidal habitat (Airoldi and Beck 2007). On the contrary, the Thesen marina was developed across an entire polluted brownfield terrestrial site, an original sand-bank island (Paarden Island) that had been purchased in 1904 to locate a sawmill and timber processing plant which it supported for almost a century, the island being renamed Thesen Island after the timber and ship-owning family concerned (Mulder 2008). The through-flow artificial canal system created on its decontamination from toxic substances used in wood processing (As, Cr, Cu, creosote tars, etc.) and redevelopment into a series of 19 small interlinked islands (Knynsa Museums 2022) was therefore a net gain to the estuarine bay’s subtidal aquatic habitat.

This may be unusual in the context of marina estates, but man-made coastal ponds and channels elsewhere have proved valuable habitats for the conservation of lagoonal and other coastal invertebrates (Barnes 1991; Allen et al. 1995), fish (Cavraro et al. 2021), and shorebirds (Rehfisch 1994). Most of the British coastal lagoons of conservation interest, for example, have been formed in one way or another by man, including excavated pits and scrapes (Barnes 1989; and see Herbert et al. 2018). Equivalent similar aquatic pools or channels that form small islands of high biodiversity in unlikely urbanised settings, including those of the seagrassed areas in the Thesen marina canals, could be categorised as novel ecosystems in the sense of Hobbs et al. (2009) produced by land-use change, and could potentially be the subject of ‘other effective area-based conservation measures’ (OECMs) (Pinto et al. 2021) creating, for example, soft-sediment equivalents of the ‘artificial marine micro-reserves’ of García-Gómez et al. (2015), Ostalé-Valriberas et al. (2022), etc.

To the best of our knowledge, this is the first time that the abundance and biodiversity of seagrass macrobenthos within any marina-estate system, greenfield or brownfield, has been surveyed, and hence there is no body of equivalent information from elsewhere with which it can be compared and no means of judging whether the brownfield Knysna situation is likely to be representative. Meanwhile, the appetite for coastal marina development is unlikely to decrease and the economic benefits of such schemes will often continue to be perceived as outweighing the disadvantages to coastal ecosystems, even to those of major international conservation significance. Three general features related to the present study, however, seem relevant to the future conservation of seagrass-associated biodiversity in such human-influenced areas. (i) A casual survey of developed areas of coastline suggests that the former Thesen Island at Knysna is far from being alone as a brownfield or derelict site on the terrestrial side of the land/sea interface (e.g. Leger et al. 2016; Fernandez et al. 2020); (ii) The potential advantages of small artificial coastal water bodies for conservation of shallow-water marine, lagoonal and brackish-water species seem well established and many have been incorporated into reserves of some form (Bamber 2010); and (iii) Design criteria for such artificial coastal habitats to serve as valuable areas of conservation are readily available (Bamber et al. 1993; Dafforn et al. 2015a, b) and could easily be included in the design of brownfield-estate waterways and, most importantly, in subsequent management protocols (Wauchope et al. 2022). Much is made locally of the beneficial effects of the Thesen marina canals on populations of the rare Knysna seahorse, suggesting that measures that lead to conservation of local biodiversity have good publicity value and public-relations advantages within the context of the operation of a commercial concern that has added to coastal urbanisation. Taken together these surely emphasise that, if planned, decontaminated and located appropriately (e.g. Epsilon 2001), brownfield-site marina development and conservation of coastal marine biodiversity need not be antithetical; and restored degraded areas can be of positive benefit to the local aquatic environment (e.g. Lemasson et al. 2020). In addition, any concomitantly lessened need for greenfield intertidal space would also be a reprieve for threatened shore-bird populations. The sustainable use of impacted coastlines is of major global concern (Bardos et al. 2020), and the comment of Adorjan et al. (2019) with respect to riverside development that ‘brown is the new green’ may apply equally well to the coast.

## Electronic supplementary material

Supplementary Material

## Data Availability

all new and historical data generated or analysed during this study are included in this published article and its supplementary information file.
