# Supplementary material for: Where ecologically ‘tis better to go brown than green: enhanced seagrass macrobenthic biodiversity within the canals of a brownfield coastal marina
Source: Biodivers Conserv. 2022 Aug 11;31(12):2981–97. doi: 10.1007/s10531-022-02468-9 (PMC9366803; doi:10.1007/s10531-022-02468-9)
Supplement: Supplementary file 1 — Supplementary Material [file 10531_2022_2468_MOESM1_ESM.pdf]

SUPPLEMENTARY DATA; Barnes RSK, Claassens L & Seath J 2022
Where ecologically 'tis better to go brown than green: Enhanced seagrass macrobenthic biodiversity within the canals of a brownfield coastal marina.
Biodiversity and Conservation

Numbers per 0.09 m^2 site of subtidal macrobenthic morphotaxa associated with *Zostera capensis* at two natural-channel and one artificial marina-canal localities in the outer (marine embayment) section of Knysna estuarine bay, Western Cape, S Africa (identifications are unconfirmed)

locality<br>site	Brenton<br>1	Brenton<br>2	Steenbok<br>1	Steenbok<br>2	Thesen<br>1	Thesen<br>2
	34º03'32"S<br>23º02'03"E	34º03'35"S<br>23º02'06"E	34º03'42"S<br>23º02'57"E	34º03'40"S<br>23º02'59"E	34º02'58"S<br>23º02'51"E	34º02'59"S<br>23º02'57"E
Polyclad 1	0	0	2	1	4	3
Polyclad 2	0	0	0	0	0	1
Thysanozoon	1	0	0	0	0	0
Nemertine 1	0	0	0	0	1	1
Nemertine 2	0	0	1	0	0	0
Nemertine 3	2	0	0	0	0	0
Tubificid	7	7	15	11	4	8
Harmothoid	0	0	0	0	0	1
Sthenelais	0	1	0	2	0	1
Sigalionid	0	0	0	2	3	2
Phyllodocid 1	0	0	1	0	0	0
Phyllodocid 2	1	0	1	0	1	0
Phyllodocid 3	1	0	0	0	0	0
Phyllodocid 4	0	0	0	1	0	0
Nereid 1	6	6	5	0	0	4
Nereid 2	0	0	0	0	2	15*
Nereid 3	0	1	1	0	1	45*
Nephtys	0	0	0	1	0	1
Syllid 1	1	0	0	0	0	2
Syllid 2	0	0	1	0	0	1
Syllid 3	0	0	0	0	0	1
Glycera	3	1	1	1	1	0
? Eurythoe	0	0	0	0	0	1
Diopatra	0	0	1	2	0	0
Marphysa	0	0	5	1	0	0
Lysidice	0	0	1	0	0	0
Scoletoma	15	11	4	10	9	45
Caulleriella	0	0	2	0	0	0
Capitella	1	0	20	0	2	0
Notomastus	7	2	0	2	12	1
Euclymene	0	0	0	0	0	1
Orbinia	11	13	7	7	18	20
Paradoneis	34	2	15	1	1	0

Spiophanes	4	0	0	0	0	0
Aonides	0	0	0	0	1	2
Prionospio	9	0	0	0	0	0
Paraprionospio	2	1	0	0	0	0
Polydora	7	0	1	0	3	0
Pseudopolydora 1	3	0	3	0	3	2
Pseudopolydora 2	0	0	2	0	0	0
Lagis	0	0	0	0	1	1
Piromis	0	0	0	0	1	0
Branchiomma	1	6	0	0	0	4
Desdemona	0	1	1	0	0	0
Pseudofabricia	36	17	0	0	0	1
Flabelligera	0	1	0	0	0	0
Cylindroleberis	33	85	9	32	157	32
Iphinoe	0	0	1	0	0	0
Dynamenella	0	1	0	2	13	8
Exosphaeroma	0	0	1	1	0	0
Paracerceis	0	0	0	0	0	3
Paridotea	8	18	32	11	5	9
Joeropsis	0	0	1	0	0	0
Ianiropsis	0	11	9	0	8	5
Ampelisca	0	7	0	0	0	0
Cymadusa	0	7	5	0	0	0
?Nototropis	0	0	1	0	0	0
Grandidierella	6	67	46	9	6	1
cf Bemblos	7	9	0	11	32	30
Hyale	0	7	0	0	0	0
Paramoera	0	0	1	3	9	4
Ericthonius	0	0	18	19	211	14
Jassa	0	0	0	0	16	0
Victoriopisa	0	1	0	0	0	0
Cyproidea	0	0	0	2	1	0
Monocorophium	0	0	0	2	16	34
Perioculodes	0	0	1	0	10	3
Caprella	0	0	3	2	3	3
Nebalia	0	0	0	1	0	0
Leptochelia	0	6	0	0	1	0
Diogenes	2	2	8	0	0	0
Hymenosoma	7	3	19	9	10	5
Danielella	3	3	0	0	0	0
Paratylodiplax	0	3	0	0	8	46
Smaragdia	0	0	2	4	2	0
Tricolia	0	0	0	0	1	0
Cerithiopsis	0	0	0	0	1	0
Alvania	0	0	0	0	3	0
Assiminea'	218	69	7	1	0	0
Alaba	2384	2116	181	3342	2634	2764
Turritella	134	13	0	0	15	9

Crepidula	0	0	0	0	1	0
Tectonatica	1	0	0	0	9	0
Nassarius	35	13	10	0	29	51
Gibbula	5	4	7	22	118	141
Mitrella	0	0	0	1	0	1
Anarchis	0	0	0	0	1	0
Indothais	1	0	0	0	0	0
Elysia	0	0	0	0	0	1
Bursatella	2	0	0	1	0	1
Aplysia	0	0	1	0	0	0
Philine	0	0	0	0	3	0
Haminoea	0	0	0	0	0	1
Retusa	0	0	0	0	0	1
Acteocina	0	0	0	0	0	3
Polycera	0	0	0	0	0	3
Favorinus	3	3	1	1	3	11
Godiva	0	0	1	0	7	0
Doris	0	1	0	0	0	0
Fulvia	1	0	0	9	0	0
Limaria	0	0	0	1	2	2
Tellina	0	0	0	4	13	4
Lasaea	0	0	0	0	0	1
Gafrarium	0	0	0	0	28	9
? Musculus	0	0	0	0	0	2
Arcuatula	3	2	0	0	5	3
Kellia	0	1	0	1	0	1
Pitar	0	0	0	1	0	0
Dosinia	1	0	2	0	0	0
Loripes	0	0	0	0	2	0
Amphipholis	1	7	0	0	11	0
Parvulastra	0	0	0	0	3	15
Marthasterias	0	0	0	0	1	0
Parechinus	0	2	1	1	4	7
Echinocardum	0	0	0	0	0	1
Leptosynapta	0	3	2	1	6	1
**nos individuals**	3007	2534	460	3536	3476	3394
**nos taxa**	40	41	47	40	58	61

*The large number of nereids present at the Thesen 2 site were all in a single 1 of the 16 component core samples

Corresponding author: RSK Barnes, rsb1001@cam.ac.uk
